# Efficient generation of HPLC and FTIR data for quality assessment using time series generation model: a case study on Tibetan medicine Shilajit

**DOI:** 10.3389/fphar.2024.1503508

**Published:** 2024-11-18

**Authors:** Rong Ding, Shiqi He, Xuemei Wu, Liwen Zhong, Guopeng Chen, Rui Gu

**Affiliations:** ^1^ State Key Laboratory of Southwestern Chinese Medicine Resources, School of Ethnic Medicine, Chengdu University of Traditional Chinese Medicine, Chengdu, China; ^2^ School of Pharmacy, Chengdu University of Traditional Chinese Medicine, Chengdu, China

**Keywords:** Shilajit, FTIR, HPLC, time series generation, classification

## Abstract

**Background:**

The scarcity and preciousness of plateau characteristic medicinal plants pose a significant challenge in obtaining sufficient quantities of experimental samples for quality evaluation. Insufficient sample sizes often lead to ambiguous and questionable quality assessments and suboptimal performance in pattern recognition. Shilajit, a popular Tibetan medicine, is harvested from high altitudes above 2000 m, making it difficult to obtain. Additionally, the complex geographical environment results in low uniformity of Shilajit quality.

**Methods:**

To address these challenges, this study employed a deep learning model, time vector quantization variational auto- encoder (TimeVQVAE), to generate data matrices based on chromatographic and spectral for different grades of Shilajit, thereby increasing in the amount of data. Partial least squares discriminant analysis (PLS-DA) was used to identify three grades of Shilajit samples based on original, generated, and combined data.

**Results:**

Compared with the originally generated high performance liquid chromatography (HPLC) and Fourier transform infrared spectroscopy (FTIR) data, the data generated by TimeVQVAE effectively preserved the chemical profile. In the test set, the average matrices for HPLC, FTIR, and combined data increased by 32.2%, 15.9%, and 23.0%, respectively. On the real test data, the PLS-DA model’s classification accuracy initially reached a maximum of 0.7905. However, after incorporating TimeVQVAE-generated data, the accuracy significantly improved, reaching 0.9442 in the test set. Additionally, the PLS-DA model trained with the fused data showed enhanced stability.

**Conclusion:**

This study offers a novel and effective approach for researching medicinal materials with small sample sizes, and addresses the limitations of improving model performance through data augmentation strategies.

## 1 Introduction

Shilajit (Zhaxun), as a traditional folk medicine, has been used for over 3,000 years and is widely used in countries such as Russia, India, Nepal, Egypt, Norway, Pakistan, and others (Agarwal et al., 2007). In China, Shilajit is an ancient traditional Tibetan medicine and is mainly distributed in the Tibetan regions of Tibet, Qinghai, and Sichuan, including Aba, Ganzi, and Liangshan at altitude of 2000 to 4,000 m (Ding et al., 2020). Previous studies have shown that Shilajit is primarily composed of animal feces, soil, impurities and organic matter (Kamgar et al., 2023). The unique geographical environment of Shilajit leads to variations in its chemical composition. Most researchers have indicated that humus components may be the dominant constituent, followed by amino acids, proteins, fatty acids, caffeic acid, gallic acid, and other bioactive compounds (Kamgar et al., 2023). Modern pharmacological studies have systematically validated various therapeutic properties of Shilajit, including anti-inflammatory, antioxidant, immunomodulatory, anti-tumor, anti-ulcer, and anti-viral effects (Bhavsar et al., 2016). Clinical trials have shown that Shilajit’s water extracts are safe and can serve as dietary supplements to enhance and regulate collagen levels in various tissues of healthy adults (Das et al., 2016; Cesur et al., 2019). Therefore, the consumption of Shilajit is increasing. Traditionally, Shilajit was classified into three grades based on the appearance (Kamgar et al., 2023). The first grade is considered to be of high quality, characterized by a black color, heavy texture, and minimal feces content. The third grade usually contains more fecal grains, and its quality is lower. Those between grades I and III are classified as the second degree.

Until now, the quality control standard for Shilajit has not been established due to its complex chemical composition and multiple sources (Wilson et al., 2011). However, with the development of analytical techniques and statistical approaches, spectral and chromatographic techniques have been employed to reveal the chemical profile of Shilajit (Tong et al., 2014; Cao et al., 2015). For example, X-ray fluorescence (XRF) has been used to reveal the elemental differences between raw Shilajit and Shilajatu Vatik (Shakya and Mohapatra, 2020). While XRF is highly effective for identifying elemental compositions and quantifying metal content, it lacks the ability to provide molecular information. In contrast, infrared spectroscopy, though it is less effective for detecting individual elements, excels in analyzing molecular structures and functional groups, making it suitable for distinguishing organic compounds and detecting impurities. Lee et al. (2021) utilized HPLC-UV to simultaneously evaluate the phenolic compound content in Shilajit, the isolated compounds showed promising bioactivity, and the validated HPLC method was used to quantify these compounds, suggesting that they are standard markers for Shilajit quality control. In our previous studies, Fourier transform infrared spectroscopy (FTIR) and near-infrared spectroscopy (NIR) combined with statistical methods have been used to classify different grades of Shilajit due to their outstanding advantages of being green, rapid, and non-destructive (Zhao et al., 2018; Li et al., 2023). Both results showed the same conclusion that substitutes could be distinguished from other grades, while the difference among grades I, II, and III was not significant. Therefore, the unique analytical methods used to evaluate the quality of Shilajit exhibited limitations in revealing the chemical profile.

In recent years, the emergence of data fusion strategies has significantly addressed the limitations of single analytical methods for quality evaluation, and has been widely employed in food quality authentication (Borràs et al., 2015). In the field of traditional Chinese medicine (TCM), multiple-level data fusion technologies have been used in quality assessment, enhancing pattern recognition and property parameters estimation (Ding et al., 2023). For instance, Wu et al. (2018) classified samples of *Paris polyphylla* using pattern recognition models integrated with a mid-level data fusion strategy, achieving accuracies of 96% and 100% in training and testing sets, respectively. In the authentication of TCM, multiple data from electronic nose, electronic tongue, electronic eye sensors, and NIR were used to accurately determine the authentic species of *Fritillariae cirrhosae* using mid-level data fusion strategies, the accuracy of the authenticity and species identification models reached 98.75% and 97.50%, respectively. (Gui et al., 2023). Additionally, data fusion strategies are widely used in research to identify the origins, years, and harvesting periods of TCM (Wu et al., 2019; Zhang et al., 2021; Li et al., 2024). It is noteworthy that the performance of chemometric models based on data fusion strategies is influenced by data pre-processing methods, algorithm types, feature extraction methods, and especially the number of samples. Specifically, the robustness of models established with few samples is difficult to assess due to insufficient elucidation of the similarities and differences among samples (Zhou et al., 2020).

The time series generation (TSG) model has been developed and widely used to generate difficult and limited data, such as electrocardiographs and financial stocks. (Koivisto et al., 2019; Wiese et al., 2020; Adib et al., 2023). Currently, mainstream TSG data are generated with the architectural combination of Recurrent Neural Network (RNN) and Generative Adversarial Network (GAN) (Esteban et al., 2017; Yoon et al., 2019; Liao et al., 2020). However, these methods cannot effectively generate long time series (TS) data because RNN models have limitations in processing inputs that are widely separated in time (Vaswani et al., 2017; Han et al., 2021). To address this, a time series data processing method using a variational autoencoder (VAE) model combined with vector quantization (VQ) (TimeVQVAE) has been proposed as a suitable alternative to overcome the limitations of TSG (Lee et al., 2023). In this model, VQ modeling is separated into low-frequency (LF) and high-frequency (HF) components. The LF component first defines the overall shape, and the HF component then fills in the details. In other words, using TimeVQVAE method to increase the experimental sample size can amplify information differences among samples. Therefore, adopting this method to expand the number of experimental samples can enhance the robustness of the classification model.

In this study, 137 batches of Shilajit samples from three grades were analyzed using FTIR and high performance liquid chromatography (HPLC). Subsequently, the unsupervised learning method of PCA was used to roughly assess the differences among the samples from different grades. Partial least squares discriminant analysis (PLS-DA) was employed to differentiate the Shilajit samples of three grades based on initial FTIR and HPLC data, generated data obtained through TimeVQVAE, and a low-level data fusion strategy. This study aimed to develop a rapid identification method for Shilajit, which is difficult to identify, by employing a data generation technique combined with pattern recognition and data fusion strategy.

## 2 Materials and methods

### 2.1 Sample preparation and pre-treatment

A total of 137 batches of Shilajit samples were collected from three sources: self-collection, Tibetan hospitals, and herbal markets. The detailed information on samples was provided in Supplementary Table S1. After collection, the samples were graded based on traditional methods and clinical experience in Tibetan medicine. The grading criteria included the darkness of the Shilajit, its density, and the presence of fecal particles, with higher quality indicated by a darker color, greater weight, and fewer fecal particles. Each criterion was scored out of 10, with a total score above 20 classified as high quality (H), scores between 10 and 20 classified as medium quality (M), and scores below 10 classified as low quality (L). The grading was conducted by Dr. Jiang Yong Silang and Professor Gu Rui from the Institute of Ethnic Medicine, Chengdu University of Traditional Chinese Medicine. The final grading resulted in 44 batches of H-grade, 60 batches of M-grade, and 33 batches of L-grade samples (Supplementary Table S1).

After grading, 100 g of Shilajit medicinal material was dissolved in 800 mL of boiling water, filtered, and this process was repeated three times. The supernatants were combined, concentrated into an extract, freeze-dried, and ground into a fine powder for further use.

### 2.2 Multi-source information acquisition

#### 2.2.1 FTIR spectra

0.02 g of each sample extract powder was accurately weighed by electronic analytical balance and blended with 2.0 g KBr crystal evenly before pressing into tablets (Jingtong Instrument Technology Co., Ltd., Tianjin, China). The FTIR data collection conditions were as follows: (1) a scanning range of 4,000–400 cm^−1^, (2) 32 cumulative scans, and (3) each sample was continuously scanned three times. To minimize the influence of CO_2_ and H_2_O, air spectra were collected every half hour as a blank background to reduce interference. The laboratory environment was maintained at a constant temperature of 25°C and a humidity of 30% RH.

In this study, the raw FTIR spectra were pretreated using OMNIC 8.2 (Thermo Fisher Scientific, United States), which included automatic baseline correction and ordinate normalization. Then, the processed spectra were input into Python 3.9 for further pretreatment.

#### 2.2.2 HPLC fingerprint

Using the electronic analytical balance (Sartorius BP211D, Germany), 0.5 g of sample powder was precisely weighed and then dissolved in 25 mL of 50% methanol. Ultrasonic extraction was performed for 30 min, after which the solvent lost due to volatilization was supplemented. The solution was then filtered to obtain the test solution and subsequently stored at 4°C for subsequent analysis. Samples were analyzed using an Agilent 1,260 Infinity HPLC system (Agilent Technologies Inc., United States) equipped with a diode array detector. Agilent ZORBAZSB-C18 (5 μm, 4.6 × 150 mm) was used to separate the samples at a column temperature of 30°C during system operation. The organic phase used in this procedure was acetonitrile (A), and the aqueous phase was 0.1% formic acid in water (B). The injection volume was 8 µL and the elution current velocity was 0.8 mL/min. Gradient elution was conducted according to the following conditions: 0.0–5.0 min (2.0%–7.0% A), 5.0–14.0 min (7.0%–10.0% A), 14.0–27.0 min (10.0%–15.0% A), 27.0–39.0 (15.0%–20.0% A), 39.0–49.0 min (20.0%–30.0% A), 49.0–59.0 min (30.0%–45.0% A), 59.0–60.0 min (45.0%–47.0% A). The detection wavelength was 280 nm.

In this study, both methanol and acetonitrile were chromatographically pure, provided by Thermo Fisher Scientific (Massachusetts, United States). Similarly, formic acid was also chromatographically pure, and the manufacturer was Shanghai Eon Chemical Technology Co. (Shanghai, China). Purified water was produced from Hangzhou Wahaha Group Co., Ltd. (Hangzhou, China).

### 2.3 TimeVQVAE model building

#### 2.3.1 Data set production

The dataset for this study included HPLC, FTIR, and low-level data fusion HPLC-FTIR from 137 batches of Shilajit samples. In this study, no preprocessing methods were applied; raw data was used to generate the data and the pattern recognition model. To prevent data leakage, 35 batches were reserved as the validation dataset for the classification model. The remaining 102 batches were divided into training and test datasets in a proportion of 7:3.

The model used in this study was a supervised model designed to generate data corresponding to the specific labels of the samples. Therefore, data were generated for each of the three grades of Shilajit according to their respective classifications.

#### 2.3.2 Model structure

In this study, the TimeVQVAE model (Lee et al., 2023) was used to generate HPLC, FTIR, and fused HPLC-FTIR data for Shilajit extract. This model is the first to use Vector Quantization (VQ) technology to address the problem of Time Series Generation (TSG). The VQ-VAE framework forms the foundational structure of the model. Compared to Autoencoders (AE) and Variational Autoencoders (VAE), VQ-VAE produce clearer reconstructed images (Van Den Oord and Vinyals, 2017).

**FIGURE 1:**
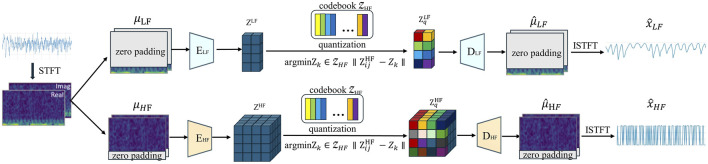
Overview of our proposed VQ (i.e., *tokenization*) (stage 1, The encoder and the decoder are denoted by E and D respectively. STFT and ISTFT stand for Short-time Fourier Transform and Inverse Short-time Fourier Transform, respectively.).

The TimeVQVAE data generation process involves two main stages. The network architecture for the first stage of the model is shown in Figure 1. Initially, the time series data is augmented to the spatio-temporal frequency domain and split into two branches: one with zero padding in the high-frequency (HF) region and the other in the low-frequency (LF) region. The E_LF_ and E_HF_ encoders then project the time-frequency domain data into a continuous latent space. During this process, each continuous label was compared to the discrete labels in the codebook using the Euclidean distance and replaced with the nearest discrete label. The decoder subsequently project the discrete latent space back into the time-frequency domain with the corresponding zero padding and maps it to the time domain via ISTFT. Finally, the two branches generate the LF and HF components of the time series.

**FIGURE 2:**
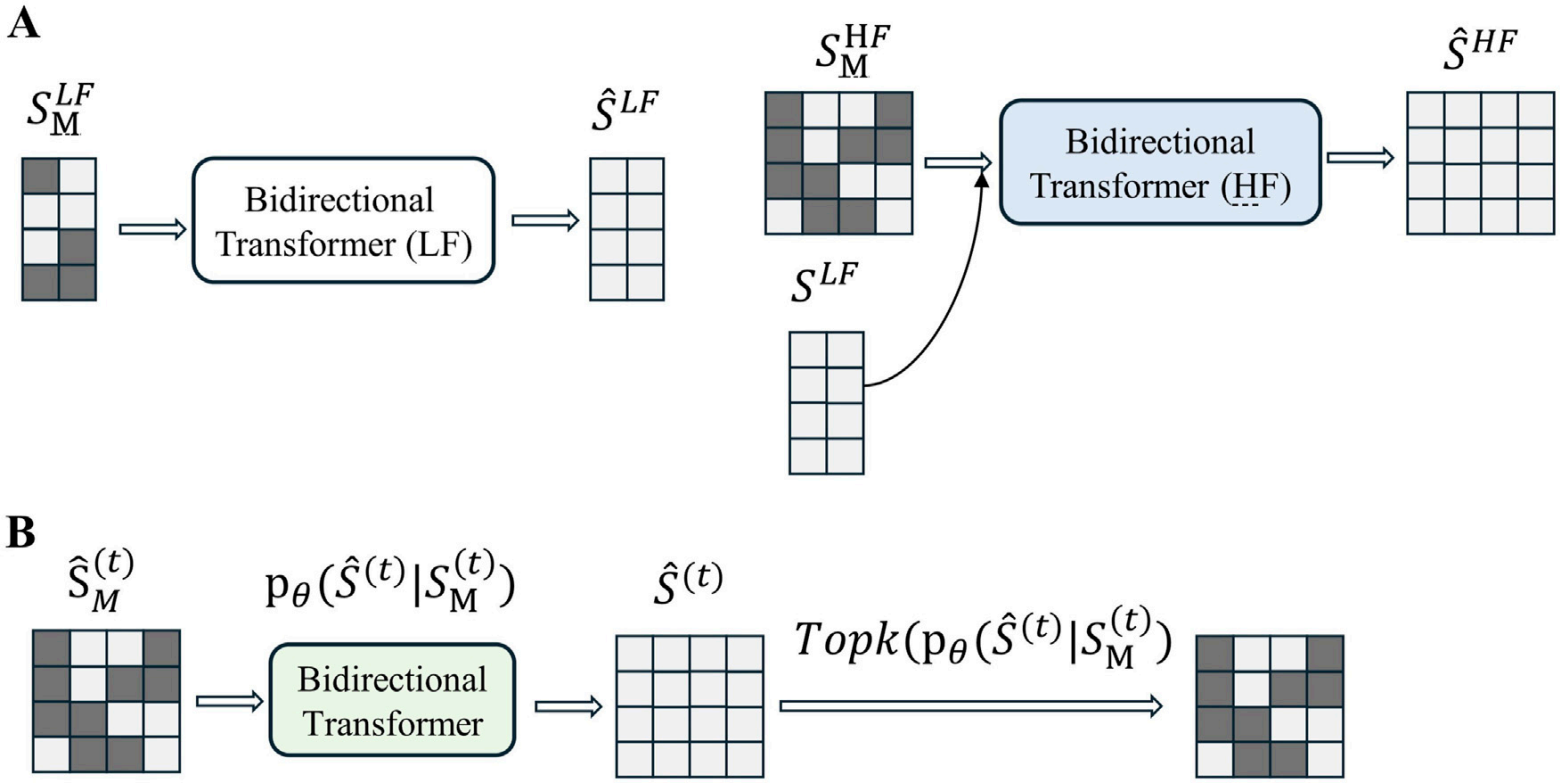
**(A)** Overview of the prior model training (stage 2). **(B)** Overview of MaskGIT’s iterative decoding. The TopK operation is equivalent to torch.topk from PyTorch. (The dark green block represents the [MASK] token.).

In the second stage, the encoder, decoder, and codebook are frozen, while the model was trained on the pre-trained discrete tokens to learn the prior, as shown in Figure 2A. Inspired by MaskGIT, a bidirectional Transformer is used as the prior model. The structure of MaskGIT within the TimeVQVAE model is shown in Figure 2B.

#### 2.3.3 Loss function

The loss function used in this study consists of two parts. The first part is the codebook loss that obtained by Equation 1:

$$L_{c\text{odebook}}={∥sg\left[E_{LF}\left(P_{LF}\left(STFT\left(x\right)\right)\right)\right]-Z_{q}^{LF}∥}_{2}^{2}+{∥sg\left[E_{HF}\left(P_{HF}\left(STFT\left(x\right)\right)\right)\right]-Z_{q}^{HF}∥}_{2}^{2}+\beta {∥\left[E_{LF}\left(P_{LF}\left(STFT\left(x\right)\right)\right)\right]-sg\left[Z_{q}^{LF}\right]∥}_{2}^{2}+\beta {∥\left[E_{HF}\left(P_{HF}\left(STFT\left(x\right)\right)\right)\right]-sg\left[Z_{q}^{HF}\right]∥}_{2}^{2}$$ (1)

Here, *x* represents the time series, sg [⋅] denotes the stop-gradient operation, P _[⋅]_ indicates the zero-padding operation for LF or HF regions, $Z_{q}^{\left[\cdot \right]}$ represents the discrete tokens for LF or HF regions, and β is the parameter for the loss term weight. Gradients are simply propagated from the decoder to the encoder through the non-differentiable quantization process.

The VQ loss also includes a reconstruction loss. In this study, the reconstruction task is performed in both the time domain and the time-frequency domain. Therefore, the formula for the reconstruction loss is shown in Equation 2:

$${\;L}_{recons}={∥x_{LF}-{\hat{x}}_{LF}∥}_{2}^{2}+{∥x_{HF}-{\hat{x}}_{HF}∥}_{2}+{∥u_{LF}-{\hat{u}}_{LF}∥}_{2}^{2}+{∥u_{HF}-{\hat{u}}_{HF}∥}_{2}^{2}$$ (2)

Here, u _[⋅]_ = P _[⋅]_(STFT(x)), where μ represents the zero-padded STFT of the original time series x , and $\hat{u}$ is the reconstruction of μ , The x _[⋅]_ and $\hat{x}$ _[⋅]_ are obtained by applying ISTFT to u _[⋅]_ and $\hat{u}$ _[⋅]_, respectively. Therefore, the final VQ loss is shown in Equation 3 :

$$L_{VQ}=L_{codebook}+L_{recons}$$ (3)

#### 2.3.4 TimeVQVAE model evaluation

In this experiment, two primary evaluation metrics were used: Inception Score (IS) (Salimans et al., 2016) and Fr´echet Inception Distance (FID) (Heusel et al., 2017). The IS ranges from 1 to the number of categories (3 in this experiment), with higher IS values indicating better quality of the generated samples. Unlike the IS, the FID score measures the quality of the generated data by comparing the distribution of generated samples to that of real samples. The FID score ranges from 0 to infinity, with lower values indicating better quality of the generated data. The formula for IS is shown in Equation 4:

$$IS\left(G\right)=\exp\;\left(E_{x∼p_{g}}D_{KL}\left(p\left(y|x\right)\|p\left(y\right)\right)\right)$$ (4)

Here, x denotes the generated samples, $p_{g}$ is the distribution of the generator, $p\left(y|x\right)$ is the conditional distribution of labels given the sample x , and $p\left(y\right)$ is the marginal distribution of labels. D*KL* represents the Kullback-Leibler divergence, and E denotes the expectation. Higher IS values indicate that the generated samples are more diverse and of higher quality. The formula for FID is shown in Equation 5:

$$FID\left(x,g\right)={\left|\left|\mu_{x}-\mu_{g}\right|\right|}_{2}^{2}+Tr\left(\sum x+\sum g-2{\left(\sum x\sum g\right)}^{0.5}\right)$$ (5)

Here, x represents the real data distribution and g represents the generated data distribution. The vectors $\mu_{x}$ and $\mu_{g}$ are the mean feature vectors of the real and generated data, respectively, extracted from the Inception v3 model. The covariance matrices of the real and generated data are denoted as ∑x and ∑g , respectively. The FID assesses the statistical differences between the real and generated data distributions in the feature space. A lower FID score indicates that the generated samples are closer to the real data distribution, suggesting higher quality and more realistic generated samples.

### 2.4 Classification model and data analysis

#### 2.4.1 Classification model

**FIGURE 3:**
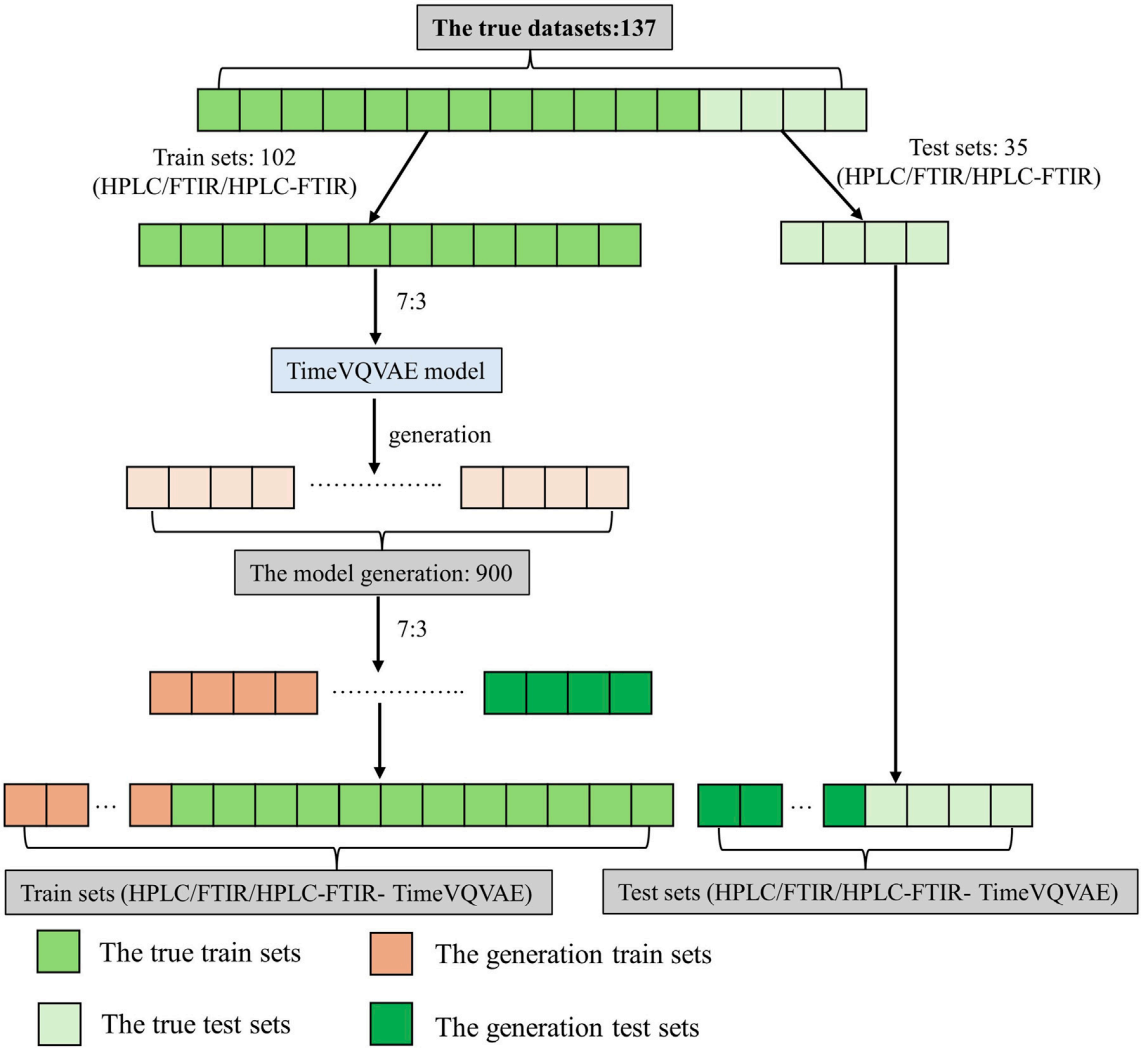
Principles of dataset partitioning.

The PLS-DA model used in this study was a classic machine learning classification model commonly used in the research of traditional Chinese medicine and food (Borràs et al., 2016; Hong et al., 2023; Deng et al., 2025). Before establishing the PLS-DA model, the TimeVQVAE generated a large number of data based on the original HPLC, FTIR, and low-level data fusion HPLC-FTIR data. The study included six types of data: (1) HPLC, (2) FTIR, (3) HPLC-FTIR (LLDF), (4) HPLC-TimeVQVAE, (5) FTIR- TimeVQVAE, (6) HPLC-FTIR- TimeVQVAE. The dataset was split using the “train_test_split” function from Python’s scikit-learn library, dividing the dataset twice, as shown in Figure 3. In this study, we employed one-hot-encoding for multi-class classification within the PLS-DA framework. This method ensured that each class was treated independently without implying any ordinal relationship (Karthiga et al., 2021; Perotti et al., 2023).

Before data generation, the training and test set for the classification model consisted of 102 and 35 samples, respectively. After data generation, the training and test set contained 732 and 305 samples, respectively. The results of data set division were shown in Supplementary Table S2.

#### 2.4.2 Classification model evaluation

In this study, the model was evaluated using four metrics: Sensitivity, Specificity, Precision, Accuracy, and F1 score (Hong et al., 2023; Szabó et al., 2024). Sensitivity, also known as the true positive rate, measures the proportion of actual positive samples correctly identified. Specificity, also known as the true negative rate, measured the proportion of actual negative samples correctly identified. Precision is defined as the number of true positive samples divided by the number of samples predicted to be positive. Higher precision indicates fewer false positives. F1 score is the harmonic mean of precision and recall, providing a balanced evaluation between these two metrics. It is used to combine precision and recall into a single measure that accounts for both metrics simultaneously. These metrics can be obtained by Equations 6–10:

$$Sensitivity\left(SEN\right)=Recall=\frac{TP}{TP+FN}$$ (6)

$$Specificity\left(SPE\right)=\frac{TN}{TN+FP}$$ (7)

$$Precision=\frac{TP}{TP+FP}$$ (8)

$$Accuracy\left(ACC\right)=\frac{TN+TP}{TN+TP+FN+FP}$$ (9)

$$F1\;score=2\times \frac{Precision\times Recall}{Precision+Recall}$$ (10)

Here, TN, TP, FN, and FP represent true negative, true positive, false negative, and false positive, respectively. TP represents the number of samples correctly classified into specific categories; TN is the number of non-specific category samples correctly assigned to non-specific categories; FP is the number of non-specific category samples belonging to specific categories; FN is the number of specific category samples incorrectly classified as non-specific categories.

### 2.5 Experimental environment and software

The experiments were conducted on a computer with the following specifications: Windows 10 operating system, Intel i9-10900X CPU, NVIDIA RTX 3090 GPU, and 96 GB of RAM. Python 3.9, PyTorch 1.8.0, and CUDA 11.8 were used for coding and executing all models. These computational resources provided sufficient power for efficient model training and evaluation.

## 3 Results and discussion

### 3.1 HPLC and FTIR fingerprints analysis

Supplementary Figure S1 displayed the HPLC fingerprint chromatograms for the three grades. Principal Component Analysis (PCA) was used to analyze the HPLC data, with results shown in Supplementary Figure S2A. The figure showed overlap between the high and medium grades, as well as between the medium and low grades, suggesting that the liquid chromatographic analysis data revealed a high degree of chemical similarity among samples with minimal differences.

Similarly, PCA analysis of the FTIR data, shown in Supplementary Figure S2B, also indicated minimal differences between the three grades, demonstrating that the grades could not be distinctly separated based on the FTIR analysis.

**FIGURE 4:**
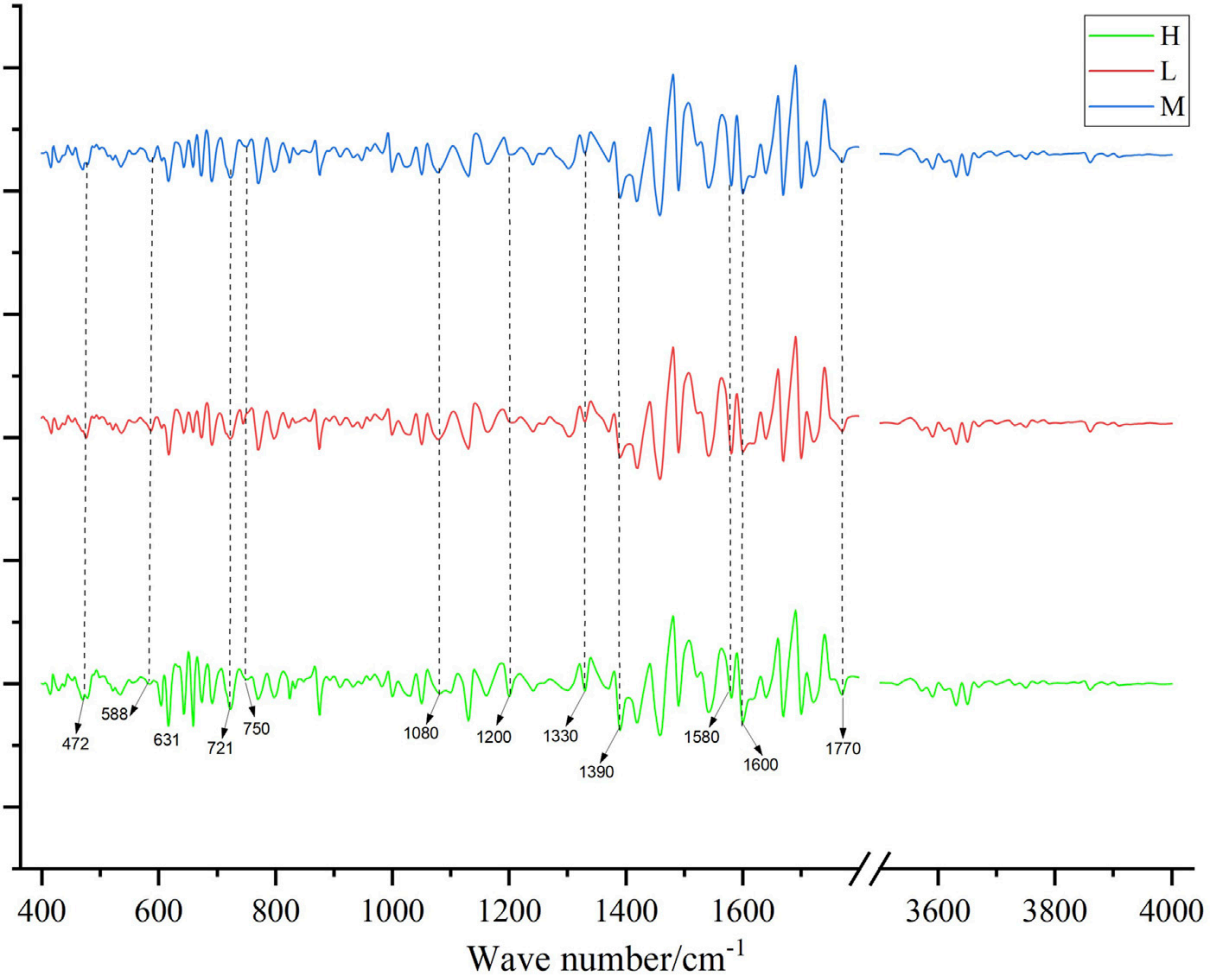
The second-order guide spectrogram of the infrared spectrum of medicinal materials, H is high quality, M is medium quality, and L is low quality.

Figure 4 displayed the average second derivative FTIR spectra for different grades. High spectral absorption between 1,390–1770 cm^−1^ is primarily attributed to the stretching vibrations of the benzene ring skeleton, carbonyl (C=O) vibrations, and C-N vibrations (Xiao et al., 2014). Peaks at 472, 588, and 631 cm^−1^ are associated with the vibrations of certain oxides or metal elements coordinated with -O or similar compounds (Justi et al., 2021). Peaks at 721 and 750 cm^−1^ are likely related to the vibrations of alkyl or alkoxy groups. Peaks at 1,080 and 1,200 cm^−1^ may be associated with C-O vibrations (e.g., carboxyl, ether), and the peak at 1,330 cm^−1^ might relate to C-N vibrations or vibrations of aliphatic aldehydes or alcohols. This suggested that Shilajit may contain conjugated benzene ring compounds and trace metal elements.

### 3.2 Data generation by TimeVQVAE model

#### 3.2.1 TimeVQVAE model training

The hyperparameters of the network were set as follows: learning rate (LR) = 0.001, weight decay = 0.00001, and the model was iterated 5,000 in total.

**FIGURE 5:**
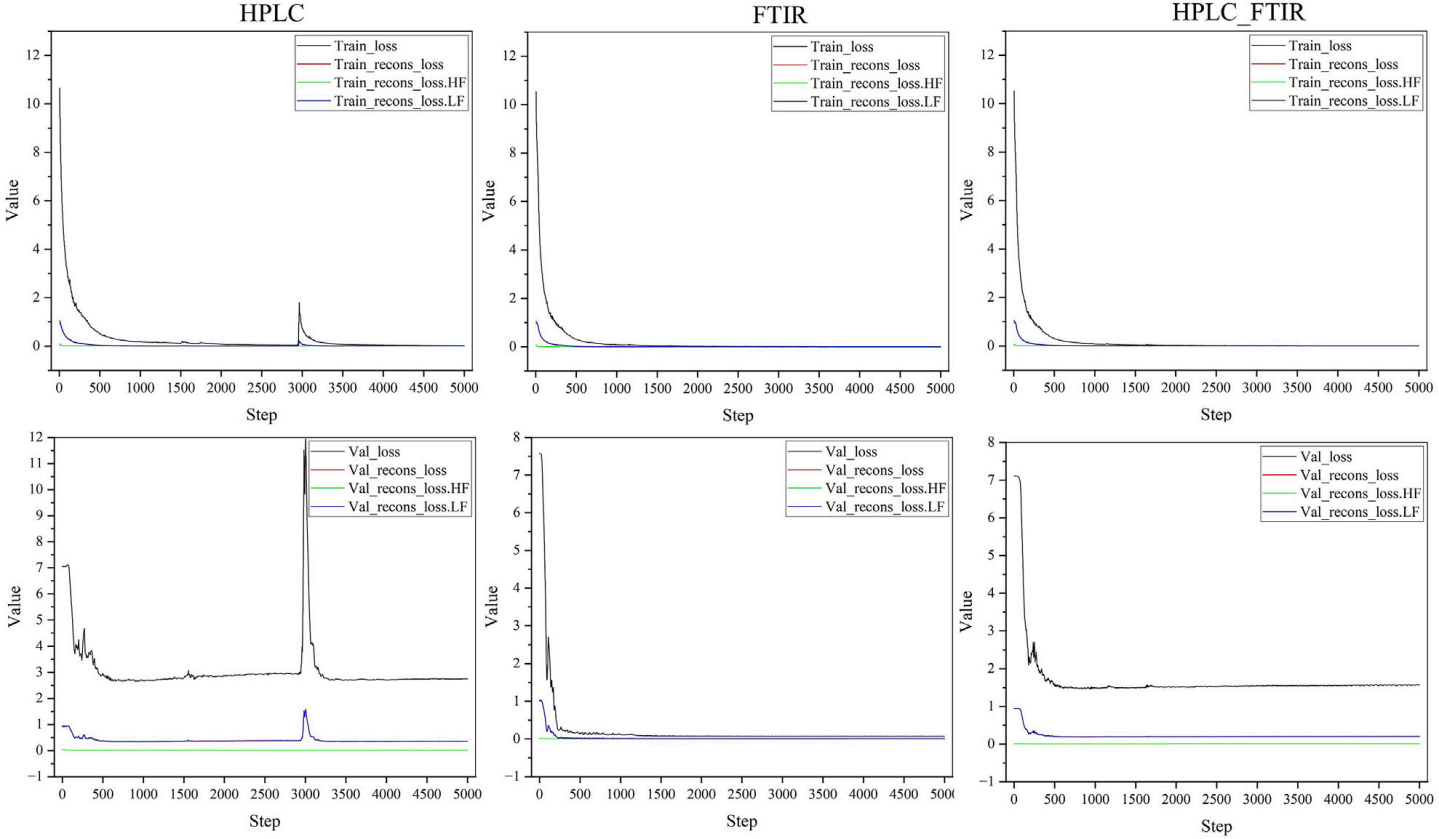
Loss variation for training and validation sets with different data sources.

Figure 5 illustrated the changes in loss during the training process. It could be observed that the loss stabilized around 4,000 iterations, with FTIR data converging the fastest. Detailed results were presented in Table 1. In the training set, losses for all three types of data were relatively low. In the test set, losses for HPLC and HPLC-FTIR were higher, with HPLC loss at 2.79325 and FTIR at 0.06987. This trend was consistent across other metrics, likely due to the higher number of features in the HPLC data, which has 9,000 feature values. After LLDF, the number of feature values increased to 10,869, resulting in higher generated data loss compared to the FTIR data.

**TABLE 1:** Final loss values for training and validation sets with different data sources.

Data type	Train set				Test set			
	Loss	Recons_loss	Recons_loss.LF	Recons_loss.HF	Loss	Recons_loss	Recons_loss.LF	Recons_loss.HF
HPLC	0.00966	0.00275	0.00014	0.00261	2.79325	0.36994	0.36133	0.00861
FTIR	0.00592	0.00157	0.00005	0.00152	0.06987	0.01242	0.00387	0.00856
HPLC_FTIR	0.00425	0.00189	0.00005	0.00184	1.57223	0.20917	0.20146	0.00771

**FIGURE 6:**
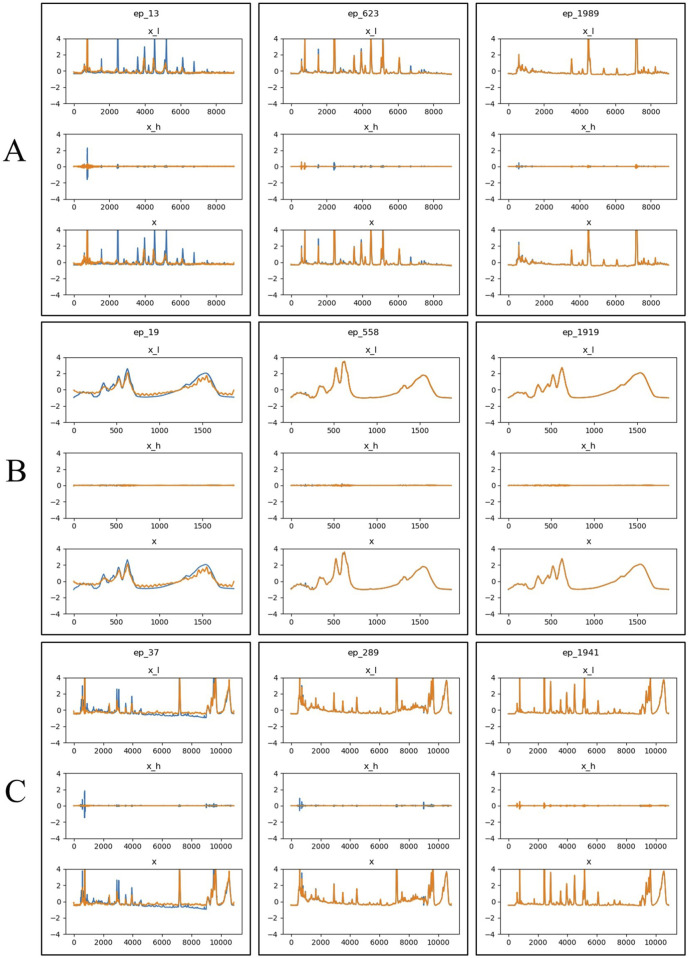
Visualization of generated results at three epochs (13, 623, and 1989) during the training process. The blue line represents the original time-series data, the yellow line represents the predicted data. x_l refers to the low-frequency component of the time-series signal, x_h refers to the high-frequency component of the time-series signal, x represents the time series data generated or predicted. **(A)** HPLC. **(B)** FTIR. **(C)** HPLC_FTIR.

Figure 6 showed the visual comparison of the original and generated data at three randomly selected epochs (13, 623, and 1989) during the training process. The blue lines represent the original input data, while the yellow lines represent the model’s predictions. As training progressed, the generated data became increasingly similar to the original data, demonstrated that the model effectively learned the key features of the original data and used this knowledge to generate new samples.

#### 3.2.2 Generated sample assessment

To fairly evaluate the generated data, robust evaluation metrics and diverse benchmark datasets are required. After training, the trained model was used to evaluate performance on the test set, with the results were presented in Table 2.

**TABLE 2:** Results of the evaluation of the three data samples.

Data type	FID	IS mean	IS std
HPLC	1.5949	1.4345	0.0731
FTIR	0.1413	1.0000	0.0000
HPLC-FTIR	3.5326	1.0791	0.0817

Table 2 showed that lower FID values and IS values closer to 3 indicate better performance. Overall, FTIR data had the smallest FID value of 0.1413 and an IS value of 1.0, indicating high quality. HPLC data also showed good performance, with an IS value of 1.4345 and an FID value of 1.5949. However, the generated data from low-level data fusion performed poorly compared to the individual datasets.

#### 3.2.3 Generate samples to visualize the assessment

**FIGURE 7:**
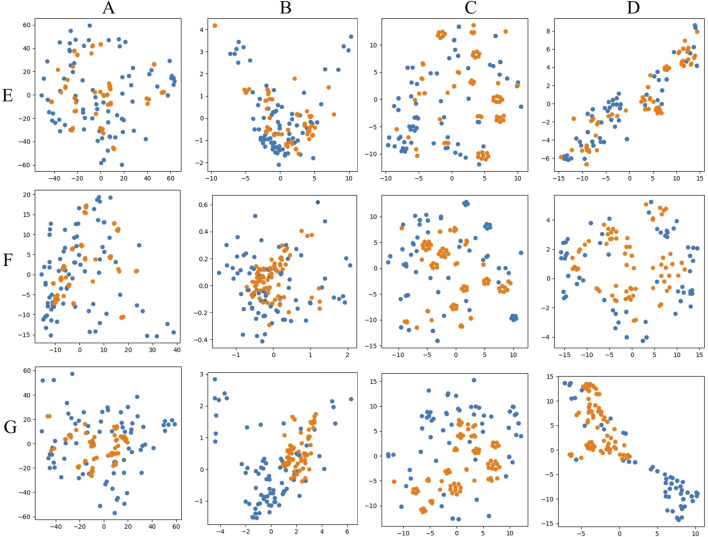
PCA and T-SNE visual mapping of test samples (blue points) and generated samples (yellow points). **(A)** PCA original data direct mapping. **(B)** PCA mapping of data in latent space. **(C)** T-SNE original data direct mapping. **(D)** T-SNE mapping of data in latent space. **(E)** HPLC. **(F)** FTIR. **(G)** HPLC_FTIR.

PCA and t-SNE were used for visual comparisons to evaluate the generated and original data, as shown in Figure 7. Figures 7A, C depicted the direct mappings of PCA and t-SNE for the original and generated data, respectively, showing that the generated data had a distribution model similar to the real data, indicating that the generation model performed well in producing samples resembling the real data. Figures 7B, D displayed the dimensionality-reduced mappings obtained by applying PCA and t-SNE in the latent space, specifically from the feature vectors extracted from a pre-trained Fully Convolutional Network (FCN) model. Here, the generated data for Figure 7E HPLC and Figure 7F FTIR exhibited distributions in the latent space that aligned closely with those of the real data, demonstrating that the model effectively captures the underlying structures and features of the data. However, Figure 7G HPLC_FTIR showed a poorer performance in the latent space, with more noticeable differences in feature distribution compared to the original data, indicating room for further optimization in capturing the combined characteristics of both datasets.

#### 3.2.4 Comparison between the generated HPLC fingerprints and the original fingerprints

**FIGURE 8:**
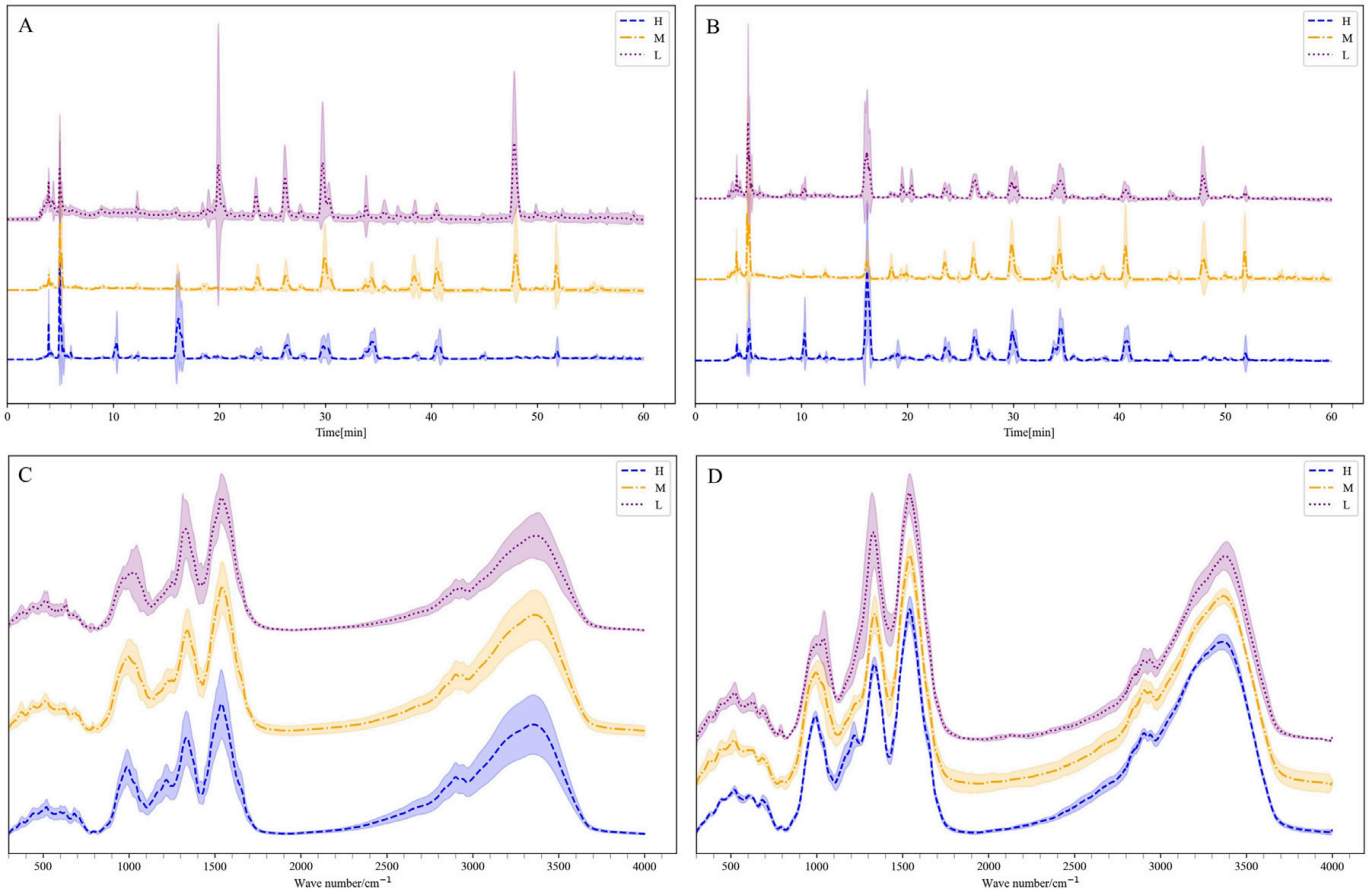
The mean and standard deviation of the generated data and the real data, H is high quality, M is medium quality, and L is low quality. **(A)** HPLC. **(B)** Generated HPLC. **(C)** FTIR. **(D)** Generated FTIR.

To better reflect the reliability of the generated data, the fingerprint chromatograms of the generated data were compared with those of the original samples. As shown in Figures 8A, B, the H grade Shilajit exhibited the highest number of peaks, followed by M and L grades. The HPLC data generated by the TimeVQVAE model showed variations in peak areas compared to the real HPLC data, with no changes in peak positions. Some individual peaks had smaller areas or were absent, which is consistent with the varying peak shapes caused by instrumental or experimental errors in traditional HPLC experiments. Moreover, the standard deviation of the generated data is much smaller than that of the real data, reducing the generation of other noise. The results demonstrate that the generated data accurately replicates the characteristics of the original data, validating the reliability and effectiveness of the TimeVQVAE model in producing high-quality HPLC data.

#### 3.2.5 Comparison between generated FTIR average spectra and the real average spectra

Supplementary Figure S3 showed the generated FTIR fingerprints spectra alongside the real spectra. It was observed from the generated and true average spectra (Figures 8C, D) that there were differences between the three grades, particularly in the range of 1,000–1,500 cm^−1^. Additionally, the average FTIR spectra generated by the TimeVQVAE model aligned closely with the true FTIR spectra for the three grades, showing a small standard deviation. This consistency indicates that the TimeVQVAE model effectively captures the characteristics of the original FTIR data, accurately reproducing the differences between the three grades.

#### 3.2.6 Generate HPLC and FTIR data

In this study, the trained TimeVQVAE model generated a total of 900 batches of data, comprising 298 batches for H grade, 311 batches for M grade, and 291 batches for L grade. The results of generated fingerprint chromatograms for the three data types were shown in Supplementary Figure S4.

### 3.3 Discriminant analyses by PLS-DA model

**FIGURE 9:**
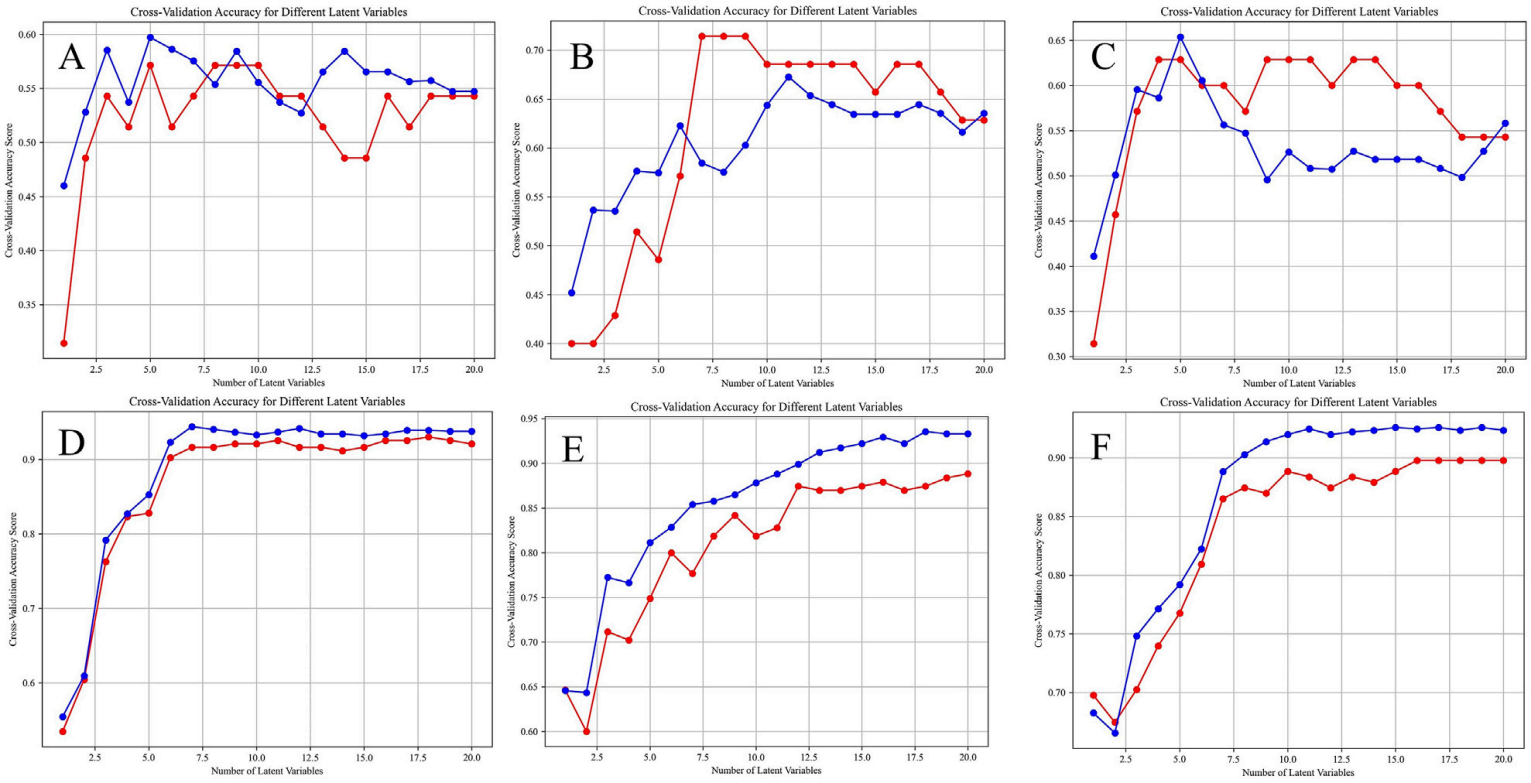
Selection of the number of latent variables through 10-Fold cross-validation, the red line and the blue line are the accuracy of the model on the training set and the average accuracy on the cross-validation set under different numbers of latent variables. **(A)** HPLC. **(B)** FTIR. **(C)** HPLC-FTIR. **(D)** HPLC-TimeVQVAE. **(E)** FTIR-TimeVQVAE. **(F)** HPLC_FTIR-TimeVQVAE.

**FIGURE 10:**
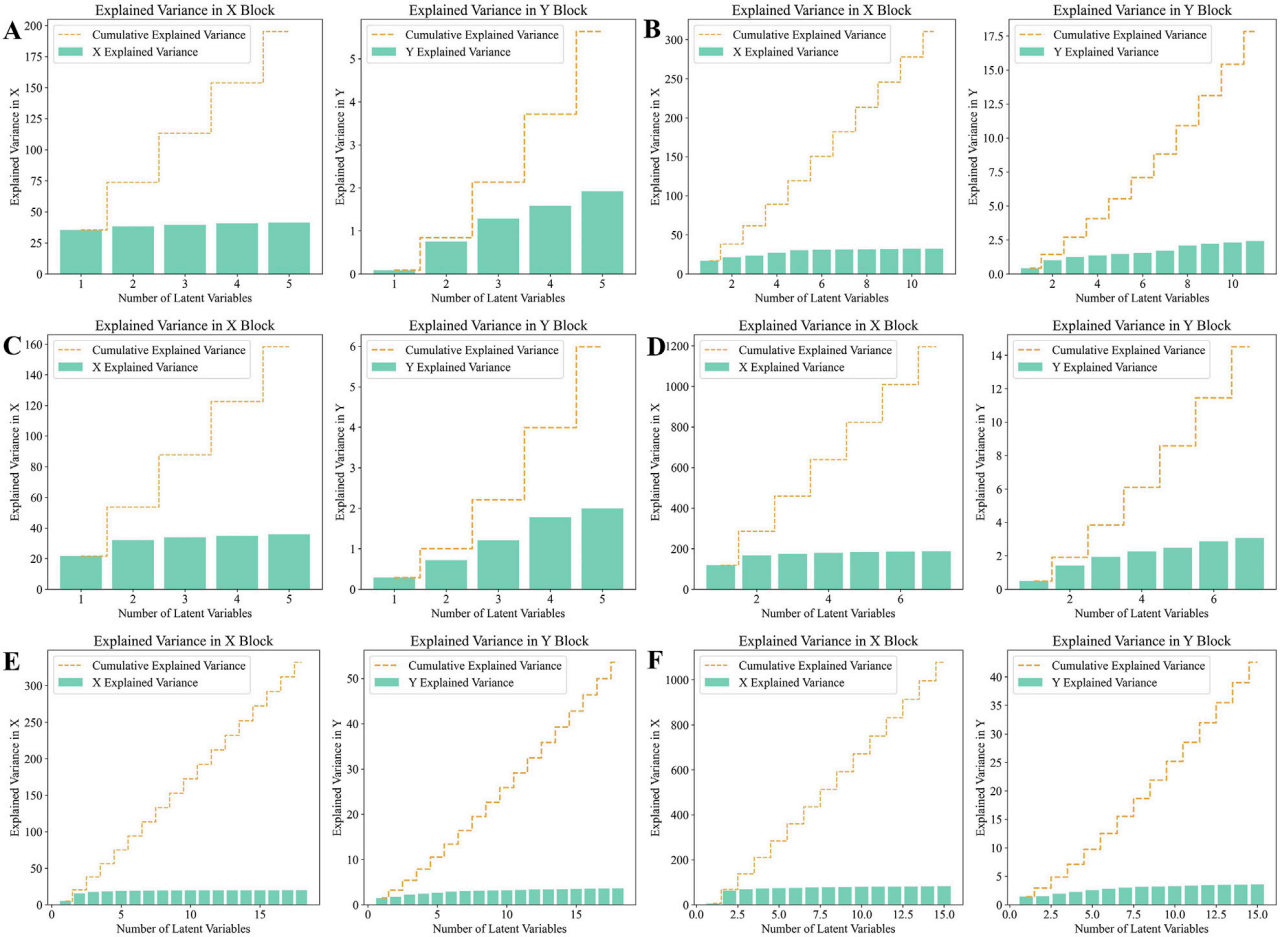
Analysis of Explained variance in X and Y Blocks in PLS-DA model. **(A)** HPLC. **(B)** FTIR. **(C)** HPLC-FTIR. **(D)** HPLC-TimeVQVAE. **(E)** FTIR-TimeVQVAE. **(F)** HPLC-FTIR-TimeVQVAE.

Before establishing the PLS-DA models, we performed 10-fold cross-validation to determine the optimal number of latent variables. The results of the selection of the number of latent variables were shown in Figure 9. Using the optimal number of latent variables determined through 10-fold cross-validation, we plotted the explained variance for both the X and Y blocks (Figure 10). The results indicated that as the number of latent variables increases, the cumulative explained variance in both blocks also increases. This suggests that the model effectively captures the variance in both the predictors (X) and the responses (Y), with a higher number of latent variables leading to better data representation. However, the rate of increase in explained variance diminishes as more latent variables are added, indicating a point of diminishing returns, beyond which additional latent variables contribute less to the model’s explanatory power.

**FIGURE 11:**
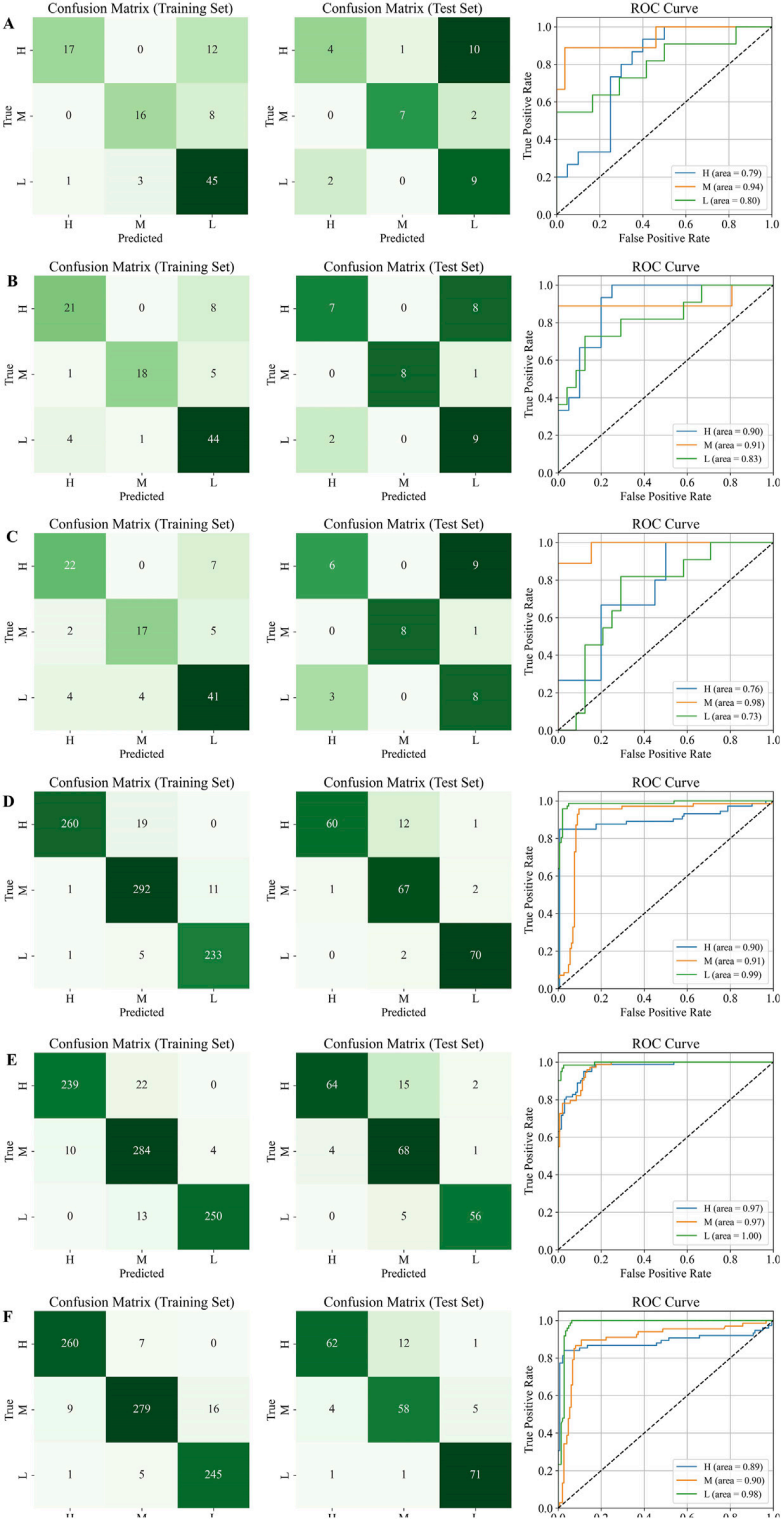
Confusion matrix and ROC curves on the test set, H is high quality, M is medium quality, and L is low quality. **(A)** HPLC. **(B)** FTIR; **(C)** HPLC-FTIR; **(D)** HPLC-TimeVQVAE; **(E)** FTIR-TimeVQVAE; **(F)** HPLC-FTIR-TimeVQVAE.

Subsequently, the optimal number of latent variables was used to build the models. The results of the confusion matrix and ROC curves were shown in Figure 11. The results showed that before data generation, the best classification performance was observed for the M grade, with significant differences in classification performance across the three grades. After virtual data generation using the TimeVQVAE model, the best classification performance shifted to the L grade. Detailed evaluation metrics are provided in Table 3.

**TABLE 3:** PLS-DA model results are based on different data types and Shilajit grades.

Data type	LVs	Grade	Train set					Test set				
			SEN	SPE	PRE	ACC	F1 score	SEN	SPE	PRE	ACC	F1 score
HPLC	5	H	0.5862	0.9863	0.9444	0.8725	0.7234	0.2667	0.9000	0.6667	0.6286	0.3810
		M	0.6667	0.9615	0.8421	0.8922	0.7441	0.7778	0.9615	0.8750	0.9143	0.8235
		L	0.9184	0.6226	0.6923	0.7647	0.7895	0.8182	0.5000	0.4286	0.6000	0.5625
		Average	0.7237	0.8568	0.8263	0.8431	0.7523	0.6209	0.7872	0.6567	0.7143	0.5890
FTIR	11	H	0.7241	0.9315	0.8077	0.8725	0.7636	0.4667	0.9000	0.7778	0.7143	0.5833
		M	0.7500	0.9872	0.9474	0.9314	0.8372	0.8889	1.0000	1.0000	0.9714	0.9412
		L	0.8980	0.7547	0.7719	0.8235	0.8302	0.8182	0.6250	0.5000	0.6857	0.6207
		Average	0.7907	0.8911	0.8423	0.8758	0.8103	0.7246	0.8417	0.7593	0.7905	0.7151
HPLC-FTIR	5	H	0.7586	0.9178	0.7857	0.8725	0.7719	0.4000	0.8500	0.6667	0.6571	0.5000
		M	0.7083	0.9487	0.8095	0.8922	0.7556	0.8889	1.0000	1.0000	0.9714	0.9412
		L	0.8367	0.7736	0.7736	0.8039	0.8039	0.7273	0.5833	0.5833	0.6286	0.5517
		Average	0.7679	0.8800	0.7896	0.8562	0.7771	0.6721	0.8111	0.7500	0.7524	0.6643
HPLC-TimeVQVAE	7	H	0.9319	0.9963	0.9924	0.9745	0.9612	0.8219	0.9930	0.9836	0.9349	0.8955
		M	0.9605	0.9537	0.9241	0.9562	0.9419	0.9571	0.9034	0.8272	0.9209	0.8874
		L	0.9749	0.9811	0.9549	0.9793	0.9648	0.9722	0.9790	0.9589	0.9767	0.9655
		Average	**0.9558**	**0.9770**	**0.9571**	**0.9700**	**0.9560**	**0.9171**	**0.9585**	**0.9232**	**0.9442**	**0.9161**
FTIR-TimeVQVAE	15	H	0.9157	0.9822	0.9598	0.9611	0.9373	0.7901	0.9701	0.9412	0.9023	0.8591
		M	0.9530	0.9332	0.8903	0.9404	0.9206	0.9315	0.8592	0.7727	0.8837	0.8447
		L	0.9506	0.9928	0.9843	0.9793	0.9671	0.9180	0.9805	0.9492	0.9628	0.9333
		Average	**0.9398**	**0.9694**	**0.9448**	**0.9603**	**0.9417**	**0.8799**	**0.9366**	**0.8877**	**0.9163**	**0.8790**
HPLC-FTIR-TimeVQVAE	15	H	0.9738	0.9820	0.9630	0.9793	0.9683	0.8267	0.9643	0.9254	0.9163	0.8732
		M	0.9178	0.9768	0.9588	0.9550	0.9378	0.8657	0.9122	0.8169	0.8977	0.8406
		L	0.9761	0.9720	0.9387	0.9732	0.9570	0.9726	0.9577	0.9221	0.9628	0.9467
		Average	**0.9559**	**0.9769**	**0.9535**	**0.9692**	**0.9544**	**0.8883**	**0.9447**	**0.8881**	**0.9256**	**0.8868**

Bold indicates the best classification result after data enhancement.

Table 3 showed that the performance of PLS-DA models built with single and fused data sources was unsatisfactory before generating data using the TimeVQVAE model. The PLS-DA model could not differentiate between the three grades of Shilajit, and there were variations in discriminatory ability and evaluation metrics among the grades. The average accuracy metrics for the three grades ranged from 0.7143 to 0.7905.

After generating data using TimeVQVAE, all performance metrics in the training set were above 0.9. In the test set, except for FTIR, the PLS-DA model performance was strong, with all metrics above 0.8. In the training set, the HPLC-TimeVQVAE model achieved the highest overall accuracy (0.9700) and F1 scores across all classes, indicating strong model performance with minimal overfitting. However, when tested on unseen data, the performance slightly declined, particularly in the H grade, suggesting some degree of overfitting to the training data. Comparatively, the FTIR-TimeVQVAE model also showed robust results, particularly in the L grade, maintaining high sensitivity and specificity in both the training and test sets. The HPLC-FTIR-TimeVQVAE combination provided balanced results across all metrics, maintaining a good trade-off between sensitivity and specificity, especially in the L grade on the test set, indicating better generalization. In summary, while all models performed well on the training data, their generalization to the test set varied. The combined HPLC-FTIR-TimeVQVAE model demonstrated the most consistent performance across both datasets, suggesting it was the most robust model for this classification task.

The PLS-DA model confirmed that the HPLC and FTIR data generated by the TimeVQVAE model significantly improved classification model performance. Compared to data fusion, which enhances the chemical information between samples, increasing the experimental sample size resulted in better classification accuracy and performance. Additionally, high performance was achieved using a single data source.

## 4 Conclusion

This study employs deep learning models of time series generation to generate virtual chromatographic (HPLC) and spectroscopic (FTIR) data, which were then used in traditional machine learning methods for classifying Tibetan medicinal Shilajit. This model not only generates a large amount of virtual data that closely resemble the original chromatographic and spectroscopic profiles but also enhances the performance and accuracy of classification models. Furthermore, for PLS-DA model trained on real samples, the average performance metrics for the PLS-DA model range from 0.5 to 0.9, therefore, both classification models struggle to distinguish among the three grades of Shilajit before using the TimeVQVAE model. However, after generating data using TimeVQVAE, the performance of the classification model significantly improved, with average classification accuracy above 0.9 for both the training and testing sets. Compared to data fusion, increasing the experimental sample size is more effective in enhancing classification model performance.

This study contributes to advances in the application of time series generation model for generating chromatographic and spectroscopic data in traditional Chinese medicine. It addresses the challenge of limited data samples and reduces the need for extensive chemical experiments by generating a large number of data that closely resemble the original data. These generated data can be considered results of instrument errors or human-induced variations during experiments. The abundance of experimental data improve the model’s robustness and accuracy.

## Data Availability

The raw data supporting the conclusions of this article will be made available by the authors, without undue reservation.
