# Supplementary material for: Efficient generation of HPLC and FTIR data for quality assessment using time series generation model: a case study on Tibetan medicine Shilajit
Source: Front Pharmacol. 2024 Nov 18;15:1503508. doi: 10.3389/fphar.2024.1503508 (PMC11608951; doi:10.3389/fphar.2024.1503508)
Supplement: Supplementary file 1 [file DataSheet1.docx]

**Supplementary Table and Figures**

**Table S1**

Information of Zhaxun Samples (H is high quality, M is medium quality, and L is low quality).

| **No.** | **Origin/source** | **Grade** | **No.** | **Origin/source** | **Grade** |
| --- | --- | --- | --- | --- | --- |
| ZX001 | Yak Gully, Danba County, Ganzi Prefecture, Sichuan Province | H | ZX078 | Derong County, Ganzi Prefecture, Sichuan Province | H |
| ZX002 | Tibetan Hospital of Xiangcheng County, Ganzi Prefecture, Sichuan Province | H | ZX079 | Hehuachi Herb Market, Chengdu, Sichuan, China | H |
| ZX003 | Hehuachi Herb Market, Chengdu, Sichuan, China | H | ZX081 | Hehuachi Herb Market, Chengdu, Sichuan, China | M |
| ZX004 | Tibet Tibet Medical College, Tibet Autonomous Region | M | ZX082 | Batang County, Ganzi Prefecture, Sichuan Province | M |
| ZX005 | Bianer Village, Muzigou, Danba County, Ganzi Prefecture, Sichuan Province, China | L | ZX083 | Hehuachi Herb Market, Chengdu, Sichuan, China | L |
| ZX006 | Hehuachi Herb Market, Chengdu, Sichuan, China | H | ZX084 | Jiulong County, Ganzi Prefecture, Sichuan Province | M |
| ZX007 | Aba County Tibetan Hospital, Aba Prefecture, Sichuan Province, China | M | ZX085 | Gaoka Village, Fubian Township, Xiaojin County, Aba Prefecture, Sichuan Province, China | M |
| ZX008 | Gaoerda Village, Malkang City, Aba Prefecture, Sichuan Province, China | H | ZX086 | Purchased by medicinal farmers in Markham, Aba Prefecture, Sichuan Province, China | M |
| ZX009 | Gansu Gannan Tibetan Medicine Hospital | M | ZX087 | Hehuachi Herb Market, Chengdu, Sichuan, China | M |
| ZX011 | Jinchuan County, Aba Prefecture, Sichuan Province | M | ZX088 | Derong County, Ganzi Prefecture, Sichuan Province | M |
| ZX012 | Hehuachi Herb Market, Chengdu, Sichuan, China | H | ZX089 | Longerjia Township, Malkang City, Aba Prefecture, Sichuan Province, China | L |
| ZX013 | Hehuachi Herb Market, Chengdu, Sichuan, China | H | ZX090 | Shannan, Tibet Autonomous Region | L |
| ZX014 | Derong County, Ganzi Prefecture, Sichuan Province | H | ZX091 | Hehuachi Herb Market, Chengdu, Sichuan, China | M |
| ZX015 | Nangqian County Tibetan Hospital, Yushu Prefecture, Qinghai Province | H | ZX092 | Caodeng Township, Malkang City, Aba Prefecture, Sichuan Province, China | M |
| ZX016 | Malcolm, Aba Prefecture, Sichuan Province | L | ZX093 | Malcolm City, Aba Prefecture, Sichuan Province | M |
| ZX017 | Hehuachi Herb Market, Chengdu, Sichuan, China | H | ZX096 | Longerjia Township, Malkang City, Aba Prefecture, Sichuan Province, China | L |
| ZX018 | Hehuachi Herb Market, Chengdu, Sichuan, China | H | ZX097 | Self-picked in Malcolm, Aba Prefecture, Sichuan Province | M |
| ZX019 | Hehuachi Herb Market, Chengdu, Sichuan, China | M | ZX098 | Aba Tibetan Hospital, Sichuan Province | M |
| ZX020 | Hehuachi Herb Market, Chengdu, Sichuan, China | H | ZX099 | Hehuachi Herb Market, Chengdu, Sichuan, China | M |
| ZX021 | Longerjia Township, Malkang City, Aba Prefecture, Sichuan Province, China | H | ZX100 | Hehuachi Herb Market, Chengdu, Sichuan, China | M |
| ZX022 | Hehuachi Herb Market, Chengdu, Sichuan, China | H | ZX101 | Hehuachi Herb Market, Chengdu, Sichuan, China | M |
| ZX023 | Caodeng Township, Malkang City, Aba Prefecture, Sichuan Province, China | H | ZX103 | Seda County Tibetan Hospital, Aba Prefecture, Sichuan Province, China | M |
| ZX024 | Kangding City, Ganzi Prefecture, Sichuan Province | M | ZX104 | Muljia Shatou Village, Jinchuan County, Aba Prefecture, Sichuan Province, China | M |
| ZX025 | Muljia Township, Jinchuan County, Aba Prefecture, Sichuan Province, China | L | ZX105 | Jukang Herbal Market, Xining City, Qinghai Province, China | M |
| ZX026 | Longerjia Township, Malkang City, Aba Prefecture, Sichuan Province, China | L | ZX107 | Lu'an Township, Aba County, Aba Prefecture, Sichuan Province, China | L |
| ZX027 | Malcolm City, Aba Prefecture, Sichuan Province | H | ZX108 | Caodeng Township, Malkang City, Aba Prefecture, Sichuan Province, China | L |
| ZX028 | Hehuachi Herb Market, Chengdu, Sichuan, China | M | ZX110 | Malcolm City, Aba Prefecture, Sichuan Province | M |
| ZX029 | Qinghai Tibetan Hospital | H | ZX112 | Seda County Tibetan Hospital, Aba Prefecture, Sichuan Province, China | L |
| ZX030 | Pushajong Township, Kangding City, Ganzi Prefecture, Sichuan Province | M | ZX113 | Hehuachi Herb Market, Chengdu, Sichuan, China | H |
| ZX031 | Daofu County, Ganzi Prefecture, Sichuan Province | H | ZX115 | Hehuachi Herb Market, Chengdu, Sichuan, China | M |
| ZX032 | Hehuachi Herb Market, Chengdu, Sichuan, China | H | ZX116 | Tibet Hospital, Tibet Autonomous Region | M |
| ZX033 | Hehuachi Herb Market, Chengdu, Sichuan, China | M | ZX118 | Caodeng Township, Malkang City, Aba Prefecture, Sichuan Province, China | L |
| ZX034 | Hehuachi Herb Market, Chengdu, Sichuan, China | M | ZX119 | Hehuachi Herb Market, Chengdu, Sichuan, China | M |
| ZX037 | Guzeng Miao Township, Muli County, Liangshan Prefecture, Sichuan Province, China | M | ZX120 | Sesmangou, Dusong Township, Jinchuan County, Aba Prefecture, Sichuan Province, China | L |
| ZX038 | Deyong County Tibetan Hospital, Ganzi Prefecture, Sichuan Province, China | H | ZX121 | Jukang Herbal Market, Xining City, Qinghai Province, China | L |
| ZX039 | Hehuachi Herb Market, Chengdu, Sichuan, China | L | ZX122 | Xining Zhuo Ma Herbs Company, Xining City, Qinghai Province, China | L |
| ZX040 | Baisong Township, Derong County, Ganzi Prefecture, Sichuan Province, China | M | ZX123 | Wholesale Department of Chinese and Tibetan Medicinal Herbs, Hehuachi Herbal Market, Chengdu, Sichuan, China | L |
| ZX041 | Purchased by medicinal farmers in Markham, Aba Prefecture, Sichuan Province, China | M | ZX124 | Hehuachi Herb Market, Chengdu, Sichuan, China | M |
| ZX042 | Hehuachi Herb Market, Chengdu, Sichuan, China | H | ZX125 | Hehuachi Herb Market, Chengdu, Sichuan, China | M |
| ZX043 | Hehuachi Herb Market, Chengdu, Sichuan, China | H | ZX126 | Muli County, Liangshan Prefecture, Sichuan Province | L |
| ZX044 | Yajiang County, Ganzi Prefecture, Sichuan Province | L | ZX127 | Jiu Long County, Hehuachi Herbal Market, Chengdu, Sichuan, China | H |
| ZX045 | Huidong County, Xichang City, Sichuan Province | M | ZX128 | Hehuachi Herb Market, Chengdu, Sichuan, China | L |
| ZX048 | Hehuachi Herb Market, Chengdu, Sichuan, China | M | ZX129 | ZhaXun paste | H |
| ZX049 | Hehuachi Herb Market, Chengdu, Sichuan, China | H | ZX130 | Hehuachi Herb Market, Chengdu, Sichuan, China | L |
| ZX050 | Kangding City, Ganzi Prefecture, Sichuan Province | M | ZX131 | Hehuachi Herb Market, Chengdu, Sichuan, China | L |
| ZX051 | Yak Gully Donggu Township, Danba County, Ganzi Prefecture, Sichuan Province | H | ZX132 | Hehuachi Herb Market, Chengdu, Sichuan, China | H |
| ZX053 | Jinchuan County, Aba Prefecture, Sichuan Province | L | ZX133 | Hehuachi Herb Market, Chengdu, Sichuan, China | H |
| ZX055 | Hehuachi Herb Market, Chengdu, Sichuan, China | M | ZX134 | Hehuachi Herb Market, Chengdu, Sichuan, China | L |
| ZX056 | Malcolm City, Aba Prefecture, Sichuan Province | M | ZX135 | Hehuachi Herb Market, Chengdu, Sichuan, China | L |
| ZX057 | Meiwogou, Huaniu Village, Xiaojin County, Aba Prefecture, Sichuan Province, China | M | ZX136 | Hehuachi Herb Market, Chengdu, Sichuan, China | H |
| ZX058 | Shili Township, Yantang County, Aba Prefecture, Sichuan Province, China | M | ZX137 | Hehuachi Herb Market, Chengdu, Sichuan, China | L |
| ZX059 | Ercha Village, Longerjia Township, Malkang City, Aba Prefecture, Sichuan Province, China | L | ZX138 | Hehuachi Herb Market, Chengdu, Sichuan, China | M |
| ZX060 | Hehuachi Herb Market, Chengdu, Sichuan, China | M | ZX139 | Hehuachi Herb Market, Chengdu, Sichuan, China | H |
| ZX061 | Hehuachi Herb Market, Chengdu, Sichuan, China | H | ZX140 | Self-picked, Caodeng Township, Malkang City, Aba Prefecture, Sichuan Province, China | H |
| ZX062 | Hehuachi Herb Market, Chengdu, Sichuan, China | H | ZX141 | Self-picked, Caodeng Township, Malkang City, Aba Prefecture, Sichuan Province, China | M |
| ZX063 | Baisong Township, Derong County, Ganzi Prefecture, Sichuan Province, China | M | ZX142 | Hehuachi Herb Market, Chengdu, Sichuan, China | L |
| ZX064 | Hehuachi Herb Market, Chengdu, Sichuan, China | M | ZX143 | Jukang Herbal Market, Xining City, Qinghai Province, China | L |
| ZX065 | Hehuachi Herb Market, Chengdu, Sichuan, China | M | ZX144 | Hehuachi Herb Market, Chengdu, Sichuan, China | M |
| ZX066 | Hehuachi Herb Market, Chengdu, Sichuan, China | H | ZX145 | Yak Gully Donggu Township, Danba County, Ganzi Prefecture, Sichuan Province | H |
| ZX067 | Mulzong Township, Jinchuan County, Aba Prefecture, Sichuan Province, China | M | ZX146 | Tianqiao Village, Jiulong County, Ganzi Prefecture, Sichuan Province, China | H |
| ZX068 | Hehuachi Herb Market, Chengdu, Sichuan, China | H | ZX148 | Hehuachi Herb Market, Chengdu, Sichuan, China | H |
| ZX069 | Muzigou, Danba County, Ganzi Prefecture, Sichuan Province, China | L | ZX149 | Sichuan Province, Chengdu City, Hehuachi Herbal Market Muli County | M |
| ZX070 | Longjia Township, Malcolm, Aba Prefecture, Sichuan Province, China | M | ZX150 | Hehuachi Herb Market, Chengdu, Sichuan, China | H |
| ZX071 | Hehuachi Herb Market, Chengdu, Sichuan, China | L | ZX151 | Liangshan Muli, Hehuachi Herb Market, Chengdu, Sichuan, China | H |
| ZX072 | Gaoka Village, Fubian Township, Xiaojin County, Aba Prefecture, Sichuan Province, China | M | ZX152 | Caodeng Township, Malkang City, Aba Prefecture, Sichuan Province, China | L |
| ZX073 | Mulzong Township, Jinchuan County, Aba Prefecture, Sichuan Province, China | M | ZX153 | Hehuachi Herb Market, Chengdu, Sichuan, China | M |
| ZX074 | Hehuachi Herb Market, Chengdu, Sichuan, China | L | ZX154 | Tibet Autonomous Region Gyumai Pun Tibetan Medicine Co. | M |
| ZX075 | Hehuachi Herb Market, Chengdu, Sichuan, China | M |  |  |  |
| ZX076 | Yak Gully, Danba County, Ganzi Prefecture, Sichuan Province, China | M |  |  |  |
| ZX077 | Yak Gully Donggu Township, Danba County, Ganzi Prefecture, Sichuan Province | H |  |  |  |

**Table S2**

Dataset partitioning results.

| **Dataset Category** | **Original Count** | **After Data Generation** |
| --- | --- | --- |
| Training Set | 102 | 732 |
| Test Set | 35 | 305 |
| Total | 137 | 1037 |


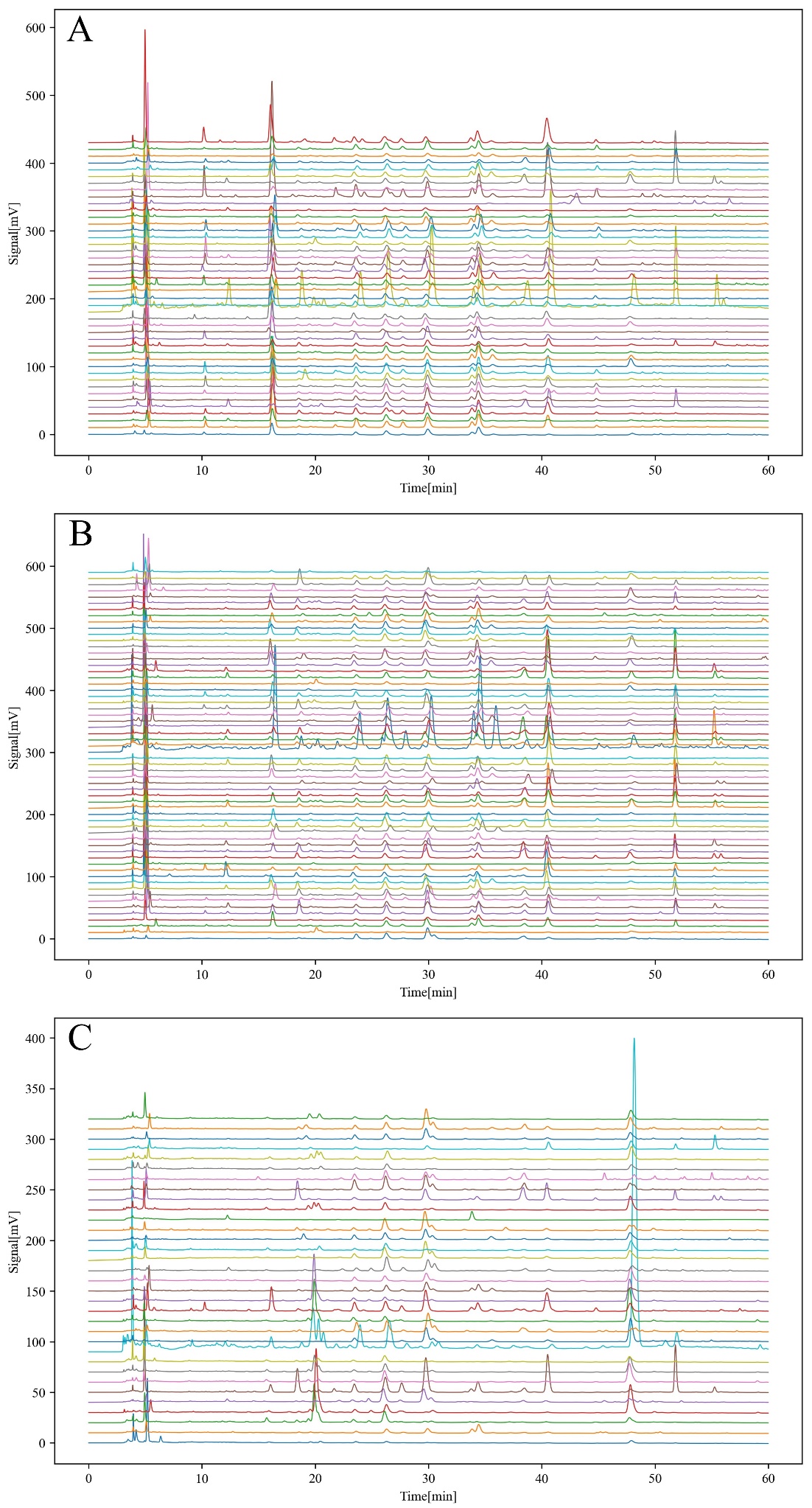


**Figure S1. HPLC fingerprints of three grades. (A: H, B: M, C: L)**


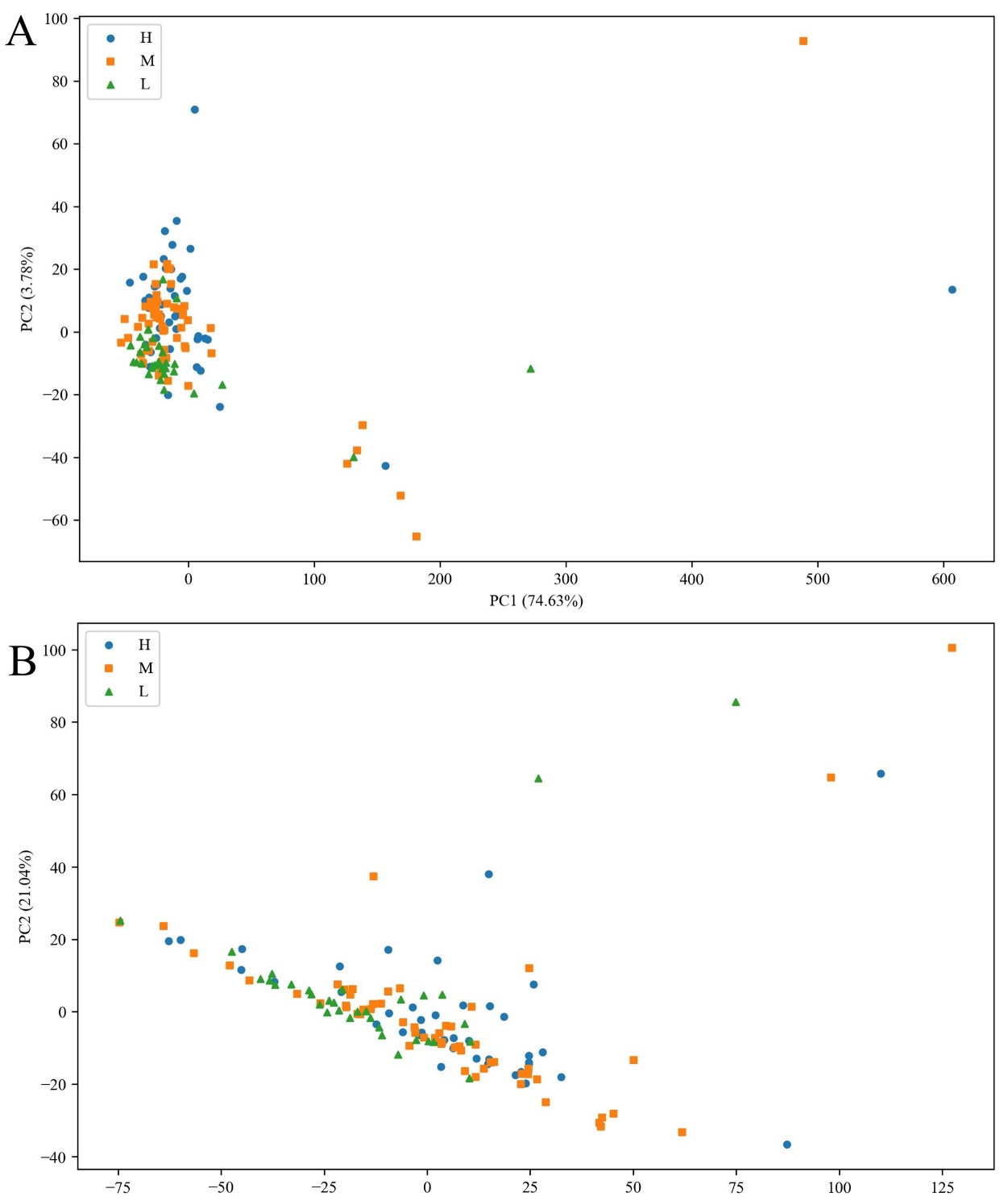


**Figure S2. PCA analysis of HPLC and FTIR fingerprint,** **H is high quality, M is medium quality, and L is low quality. (A). HPLC. (B). FTIR**


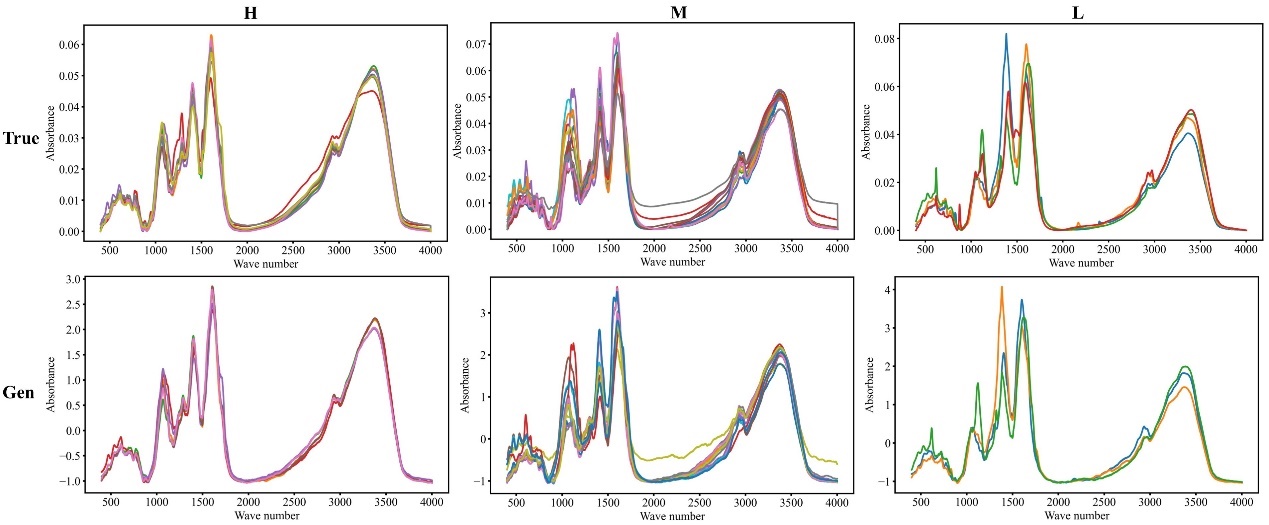


**Figure S3. Comparison of generated FTIR fingerprints and real FTIR fingerprints,** **H is high quality, M is medium quality, and L is low quality, True is the data from the real experiment, and Gen is the data generated by the TimeVQVAE model.**


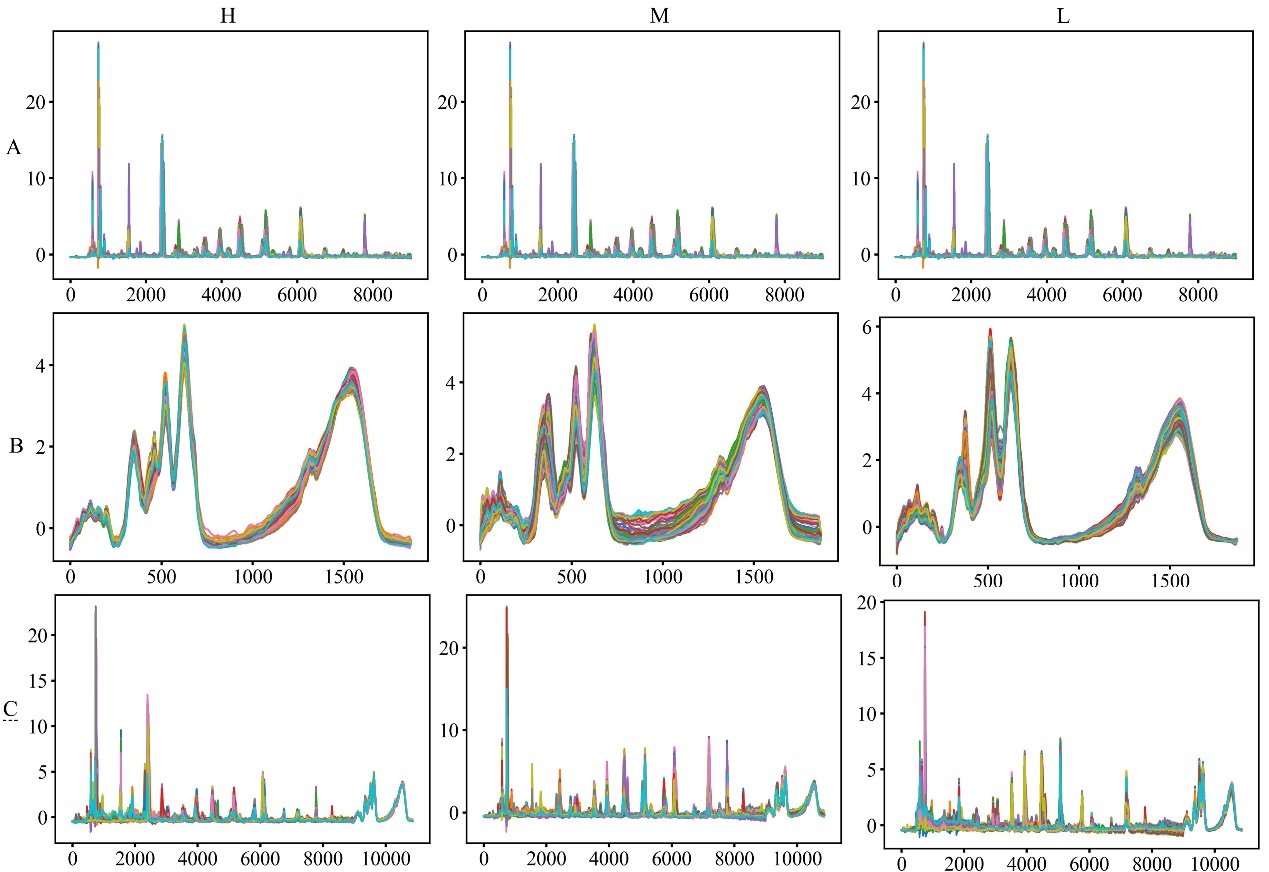


**Figure S4. TimeVQVAE-Generated 900 Fingerprint Data, H is high quality, M is medium quality, and L is low quality. (A). HPLC. (B). FTIR. (C). HPLC_FTIR.**
